# Anti-gamma-aminobutyric acid B receptor antibody-associated limbic encephalitis in relapsing polychondritis: a rare case report and literature review

**DOI:** 10.3389/fimmu.2025.1687704

**Published:** 2025-10-29

**Authors:** Jinling Zhang, Baiyu Li, Lijuan Bao, Tianjiao Zhang, Yishu Zhang, Minghua Zhang, Taowen Ren

**Affiliations:** ^1^ Department of Neurology, Gansu Provincial Hospital, Lanzhou, China; ^2^ College of Clinical Medicine, Hangzhou Normal University, Hangzhou, China

**Keywords:** relapsing polychondritis, anti-gamma-aminobutyric acid B receptor, limbic encephalitis, autoimmune mechanism, central nervous system

## Abstract

Relapsing polychondritis (RP) is an immune-mediated disorder that primarily involves the targeting of cartilaginous tissues for inflammation and destruction. Limbic encephalitis (LE) is a rare central nervous system (CNS) manifestation of RP. We report the case of a 39-year-old man who was diagnosed with RP complicated by anti-gamma-aminobutyric acid B receptor (anti-GABABR) antibody−associated LE and presented with recurrent headaches, fever, bilateral auricular swelling, scleral injection, and cognitive impairment. Laboratory tests revealed positive anti-GABABR IgG antibodies in both the serum (titer 1:100) and the cerebrospinal fluid (CSF) (titer 1:1), along with CSF lymphocytic pleocytosis. A brain MRI revealed bilateral frontal and parietal subcortical and periventricular T2-weighted fluid-attenuated inversion recovery (T2-FLAIR) hyperintensities. Immunosuppressive therapy with high-dose methylprednisolone and cyclophosphamide induced rapid symptom resolution, and no relapse occurred during a follow-up period of 1 year. This case expands the spectrum of RP-associated LE, emphasizes the necessity of neuronal autoantibody screening in RP patients with neurological symptoms, and suggests potential pathogenic links involving antigenic cross-reactivity between cartilage and neural tissues and GABAergic metabolism dysregulation.

## Introduction

Relapsing polychondritis (RP) is a rare immune-mediated disease characterized by recurrent inflammation of cartilaginous structures (mainly in the ears, nose, and tracheobronchial tree). It is also associated with various systemic features (e.g., eyes, heart, and joints) (1). Neurological involvement in RP is rare and affects only 3% of patients (2). Central nervous system (CNS) involvement may manifest as aseptic meningitis, limbic encephalitis (LE), vascular events, or cranial neuropathies (1, 2).

RP-associated LE is extremely rare, with reported cases primarily including antibody-negative LE, anti-*N*-methyl-d-aspartate receptor antibody encephalitis, and antineutral glycosphingolipid antibody-associated LE (3–8). Anti-gamma-aminobutyric acid B receptor (anti-GABABR) antibody encephalitis is a rare subtype of autoimmune encephalitis (9). We report the first documented case of RP with GABABR antibody−associated LE, suggesting a possible pathogenic overlap between cartilage-specific and neuronal autoimmunity.

## Case presentation

A 39-year-old man was admitted in July 2024 with a 1-week history of recurrent severe headaches, fever, vertigo, bilateral auricular swelling, scleral injection, and bradyphrenia. He had a medical history of hypertension. Initial symptomatic treatment (non-steroidal anti-inflammatory drugs) temporarily improved his fever and inflammation, but symptoms recurred upon cessation. The patient denied arthralgia, arthritis, respiratory symptoms (e.g., dyspnea and stridor), and laryngotracheal involvement. He had no history of chronic medication use.

**Figure 1:**
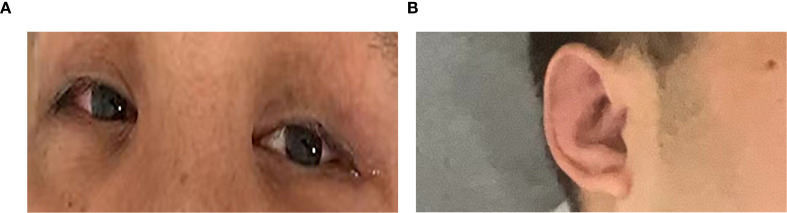
**(A)** Right scleral injection. **(B)** Mild auricular swelling. The most severe signs were not photographed.

Physical examination revealed a temperature of 37.9°C, swollen pinnae bilaterally, and right scleral injection (**Figure 1**). Neurological assessment revealed mild cognitive impairment (Montreal Cognitive Assessment (MoCA) score of 25/30, university educated) with no other neurological deficits.

**Figure 2:**
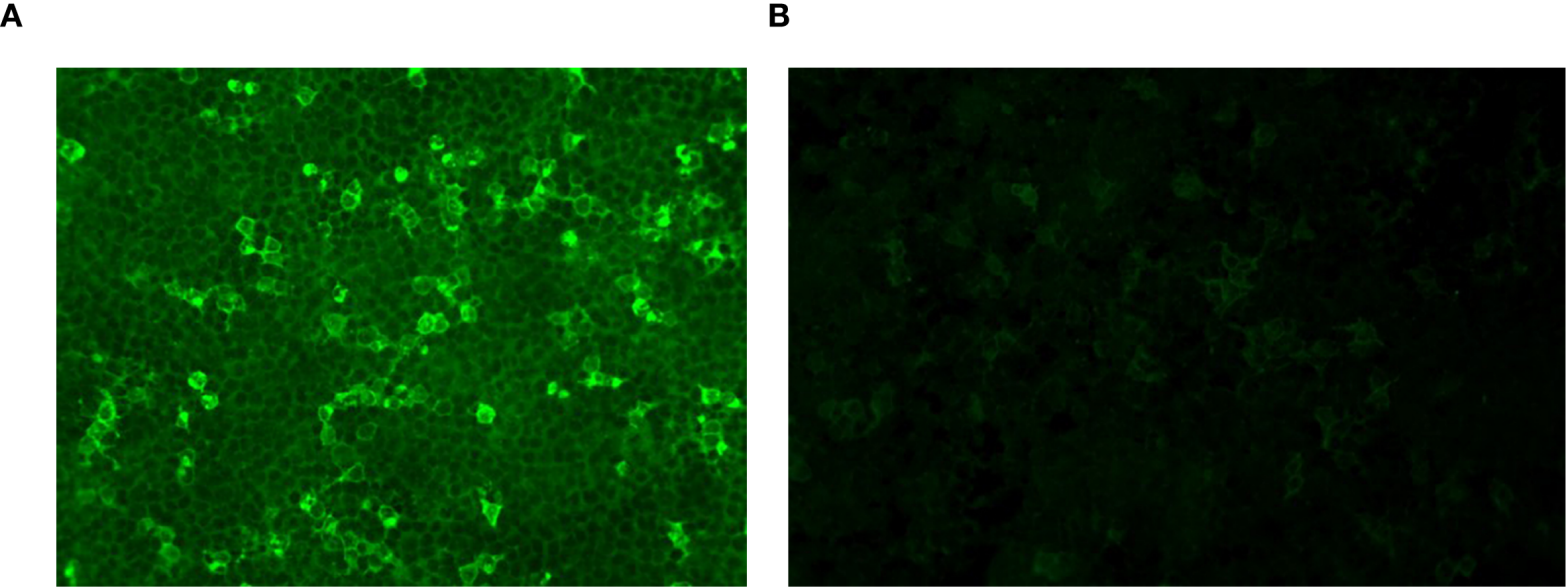
GABABR-expressing HEK293T cells were immunofluorescence-stained with serum and CSF. **(A)** Anti-GABABR IgG titer in the serum was 1:100 (×40). **(B)** Anti-GABABR IgG titer in the CSF was 1:1 (×40). GABABR, gamma-aminobutyric acid B receptor; CSF, cerebrospinal fluid.

Laboratory tests revealed increased inflammatory markers: increased serum C-reactive protein (CRP; 176.53 mg/L), leukocytosis [white blood cells (WBCs), 13.9 × 10^9^/L], and an elevated erythrocyte sedimentation rate (ESR; 47 mm/h). Serological tests excluded syphilis, human immunodeficiency virus, and Whipple disease. Rheumatoid factor and antinuclear antibodies were negative. Cerebrospinal fluid (CSF) analysis revealed elevated intracranial pressure (300 mmH_2_O), white cell count (268/μL; 96% lymphocytes), protein (0.93 g/L) and IgG (112 mg/L), and low glucose (2.72 mmol/l; serum glucose, 4.9 mmol/l). CSF microbial next-generation sequencing was negative. Anti-GABABR IgG was positive in both the serum (1:100) and the CSF (1:1) (**Figure 2**).

**Figure 3:**
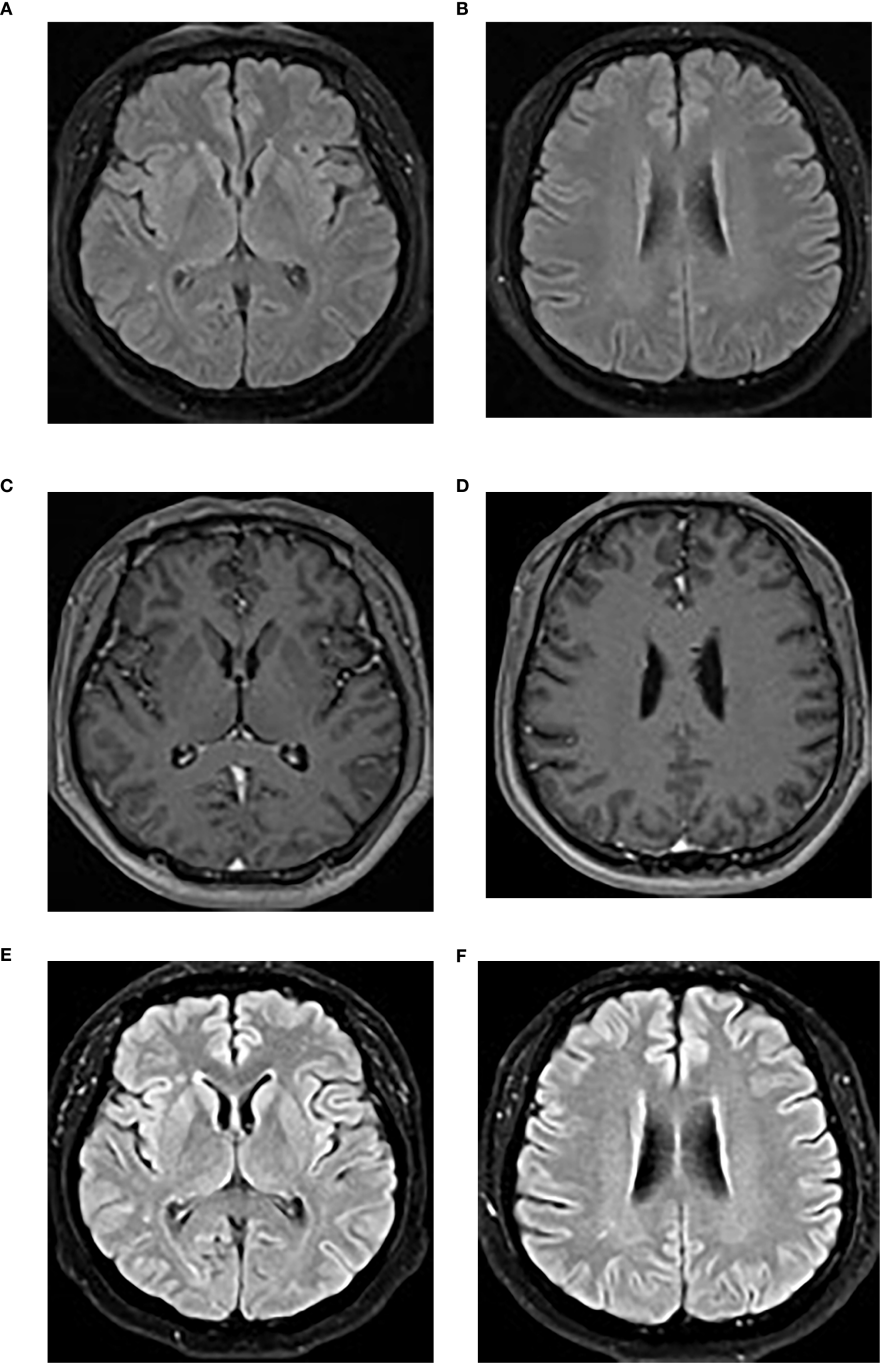
**(A, B)** The initial brain MRI image demonstrated scattered punctate T2-FLAIR hyperintensities in the bilateral frontal and parietal subcortical and periventricular regions. **(C, D)** Post-contrast T1-weighted imaging showed no abnormal enhancement. **(E, F)** A repeat brain MRI performed at the 4-month follow-up showed no significant changes on the T2-FLAIR sequence. T2-FLAIR, T2-weighted fluid-attenuated inversion recovery.

A brain MRI revealed bilateral frontal and parietal subcortical and periventricular scattered punctate lesions with T1 hypointensity, T2 hyperintensity, and T2-weighted fluid-attenuated inversion recovery (FLAIR) hyperintensity without abnormal enhancement or diffusion restriction, suggesting white matter demyelination changes (**Figure 3**).

The diagnosis of RP was based on Damiani’s modified criteria (10), with bilateral auricular chondritis, ocular inflammation, and vertigo, and a favorable response to corticosteroids, fulfilling both the clinical and therapeutic criteria. The diagnosis of anti-GABABR antibody-associated LE was supported by subacute cognitive impairment, antibody positivity in the CSF and serum, CSF pleocytosis, and the exclusion of alternative causes, fulfilling diagnostic criteria for limbic encephalitis (11).

The patient was initiated on immunosuppressive therapy with intravenous methylprednisolone (80 mg/day for 7 days), during which time an intermittent low-grade fever persisted. Cyclophosphamide was then added at a dose of 0.6 g intravenously every 2 weeks (based on a body weight of 77 kg), which led to the rapid resolution of symptoms. Methylprednisolone was subsequently switched to an oral formulation (60 mg/day) and gradually tapered until discontinuation over a total course of 8 months. Cyclophosphamide was administered for a total of 15 infusions (cumulative dose: 9 g) over a treatment period of 9.5 months.

After 23 days of immunosuppressive therapy, ^18^F-fluorodeoxyglucose (^18^F-FDG) PET/CT revealed pulmonary inflammatory micronodules (considered to be reactive inflammatory changes), but no malignancy, and electroencephalography was unremarkable. A chest CT scan performed at admission revealed micronodules, and no progression was observed in the follow-up examination after 4 months.

Over 1 year of intermittent follow-up during immunosuppression, no relapse occurred, cognitive impairment improved significantly (MoCA score 29/30), serum inflammatory markers normalized, and CSF parameters trended toward normal, with the exception of mild CSF leukocytosis (**Table 1**). A 4-month follow-up brain MRI showed no significant changes (**Figures 3E, F**). Anti-GABABR IgG titers were not reassessed due to patient refusal.

**Table 1:** Clinical symptoms, laboratory findings, and treatment details before and after immunosuppressive therapy.

Category		Timeline				Units	Normal range	Units
		7 days pre-IT	0 days IT	7 days post-IT	53 days post-IT			
Clinical symptom	Fever	Y	Y	Y	N	N/A	N/A	N/A
	Headache	Y	Y	N	N	N/A	N/A	N/A
	Vertigo	Y	Y	N	N	N/A	N/A	N/A
	Auricular swelling	Y	Y	N	N	N/A	N/A	N/A
	Scleral injection	Y	Y	N	N	N/A	N/A	N/A
	Cognitive impairment (MoCA score)	–	25	25	26	Score	26–30	Score
Serum inflammatory marker	WBC	13.9	11.8	8.9	9.3	×10^9^/L	3.5–9.5	×10^9^/L
	CRP	176.53	29.00	–	0.83	mg/L	0–6	mg/L
	ESR	–	47	26	6	mm/h	0–15	mm/h
CSF findings	Intracranial pressure	–	300	210	160	mmH_2_O	80–180	mmH_2_O
	WBCs	–	268	67	9	× 10^6^/L	0–8	× 10^6^/L
	Lymphocytes	–	96	88	0	%	N/A	%
	Protein	–	0.93	0.50	0.31	g/L	0.15–0.45	g/L
	IgG	–	112.00	73.90	21.70	mg/L	4.8–58.6	mg/L
IT	Methylprednisolone	N	80 mg/day IV	60 mg/day oral, tapered over 8 months				
	Cyclophosphamide	N	N	0.6 g IV every 2 weeks (total 15 times/9 g) over 9.5 months				

IT, immunosuppressive therapy; WBCs, white blood cells; CRP, C-reactive protein; ESR, erythrocyte sedimentation rate; CSF, cerebrospinal fluid; Y, yes; N, no; N/A, not applicable; IV, intravenous; -, no record available.

## Discussion

The pathogenesis of RP remains incompletely understood, but it is widely regarded as an immune-mediated inflammatory disease, characterized by the targeting of cartilage components, primarily type II collagen and matrix proteins (e.g., matrilin-1) (12). This process triggers both humoral immunity (production of autoantibodies) and cellular immunity, leading to the release of proinflammatory cytokines (e.g., TNF-α, IL-1β, and IFN-γ) and chemokines that recruit inflammatory cells and exacerbate tissue damage (12). Genetic susceptibility is linked to HLA-DR4, while enzymatic degradation by matrix metalloproteinases directly disrupts cartilage structure (13). Approximately 30% of patients exhibit coexisting autoimmune disorders, reflecting shared immune dysregulation mechanisms (14).

RP-associated LE is an extremely rare and severe neurological complication of RP. Currently identified types include antibody-negative LE, anti-glutamate receptor GluRϵ2 encephalitis, and anti-neutral glycosphingolipid antibody-associated LE (3–8, 15). Main CNS symptoms include cognitive dysfunction, memory impairment, psychiatric symptoms (e.g., agitation and hallucinations), and seizures. Brain MRI often shows T2-FLAIR sequence hyperintensities in the medial temporal lobes, with some cases progressing to atrophy (11). Our case lacked typical MRI features; however, early disease or atypical presentation could account for this discrepancy. PET/CT is more sensitive than an initial MRI for LE diagnosis (16). Furthermore, PET/CT can also aid in the early diagnosis of RP by assessing hypermetabolic activity in affected areas (17). Post-immunosuppressive therapy, PET/CT in this case showed no metabolic abnormality, limiting its diagnostic utility.

The pathogenesis of RP-associated LE is still unknown, although it appears to be related to autoimmunity. Mihara et al. demonstrated that RP-associated LE involves the autoimmune targeting of neutral glycosphingolipids (glucosylceramide and galactosylceramide), which are uniquely detected in patient serum. These antibodies enhance nerve growth factor-induced Trk autophosphorylation, which directly disrupts neuronal signaling in limbic regions (4). Additionally, the presence of antibodies against glutamate receptor subunits, notably GluRϵ2 (NR2B), found in the CSF and serum of certain RP-LE patients, implicates autoimmunity targeting neuronal surface antigens, potentially disrupting synaptic transmission and contributing to excitotoxicity (3, 15, 18). Stewart et al. established that CNS vascular inflammation causes ischemic injury and blood–brain barrier disruption via autopsy evidence (19). Molecular mimicry between cartilage components (e.g., type II collagen) and neuronal antigens may initiate cross-reactive autoimmunity, while vascular inflammation facilitates the penetration of antibodies into the CNS, thereby driving clinical manifestations.

Anti-GABABR antibodies are typically associated with LE. The pathogenesis of anti-GABABR encephalitis involves autoantibodies targeting the GABAB receptor, which inhibit receptor function by blocking GABA binding or receptor activation, leading to reduced inhibitory neurotransmission and neuronal hyperexcitability (20, 21). Approximately 50% of anti-GABABR LE cases are associated with malignancy, especially small-cell lung cancer. Tumor cells express GABAB receptors or related antigens, such as KCTD16, triggering antibody production through molecular mimicry (22). These antibodies may also cross-react with neuronal receptors. In non-malignancy cases, the trigger is unknown but may involve infections, other autoimmune conditions, or genetic factors (e.g., HLA associations) that lead to immune activation and antibody production (23). Antibodies are produced intrathecally and can be detected in the CSF, causing direct functional blockade of GABAergic signaling (21). PET/CT excluded malignancy in this case. To our knowledge, this is the first report of anti-GABABR antibody-associated LE in a patient with RP, implying a disease-specific pathogenic link.

One potential pathogenic link involves antigenic cross-reactivity between cartilage and neural tissues. Murine studies have confirmed that chondrocytes express GABABR, which interacts with the Ca^2+^-sensing receptor to regulate chondrocyte function and promotes ATF4 nuclear translocation for chondrogenesis (24, 25). In RP, immune attacks on cartilage disrupt GABABR, and we speculate that the structural homology between chondrocyte and neuronal GABABR induces autoantibodies. These antibodies then cross-react with neuronal GABABR, impairing GABAergic signaling and causing limbic encephalitis.

Additionally, GABAergic metabolism dysregulation may link cartilaginous and neuronal pathology. Shen J et al. (26) found that the overexpression of 4-aminobutyrate aminotransferase (Abat), a key enzyme that catalyzes the conversion of GABA to succinic semialdehyde, not only disrupts mitochondrial function and causes abnormal chondrocyte energy metabolism but also triggers inflammatory responses, further damaging cartilage. In our case, the coexistence of RP and anti-GABABR antibody encephalitis suggests that Abat dysfunction may induce GABA metabolic imbalance, which not only contributes to cartilage inflammation in RP by impairing chondrocyte energy metabolism but also reduces GABA-mediated inhibitory signaling by affecting GABABR function, ultimately triggering neuroinflammation and the clinical manifestations of limbic encephalitis.

Thus, the exact pathogenesis linking RP and anti-GABABR antibody-associated LE remains unclear and requires further verification, particularly for exploring potential common epitopes between cartilage-specific proteins and GABABR subunits, along with the role of GABAergic metabolism dysregulation.

The management of RP with CNS involvement remains challenging due to the rarity of such cases and the lack of standardized treatment protocols. Current approaches are largely empirical. High-dose glucocorticoid pulse therapy serves as the primary treatment modality. However, some case reports indicate an insufficient initial therapeutic response or a tendency to relapse when tapering is too rapid (3, 8, 27). For patients with glucocorticoid-refractory disease or those requiring long-term oral glucocorticoids, combination therapy with other agents (such as cyclophosphamide, intravenous immunoglobulin, or infliximab) may be considered (15, 28). In this case, the patient continued to experience intermittent low-grade fevers during treatment with 80 mg/day of methylprednisolone. Complete symptom resolution was achieved following the addition of cyclophosphamide, and no relapse occurred during the 1-year follow-up period.

## Conclusion

This report presents the first case of RP with anti-GABABR antibody-associated LE, expanding the spectrum of autoimmune mechanisms in RP. Antigenic cross-reactivity between cartilage and neural tissues, along with GABAergic dysregulation, represent possible mechanisms; however, these findings require further validation. Early neuronal autoantibody screening for RP patients with neurological symptoms can enable precise diagnosis and targeted immunosuppressive therapy, thereby minimizing irreversible brain tissue damage.

## Data Availability

The original contributions presented in the study are included in the article/supplementary material. Further inquiries can be directed to the corresponding author.
